# The power Lomax distribution with an application to bladder cancer data

**DOI:** 10.1186/s40064-016-3464-y

**Published:** 2016-10-21

**Authors:** El-Houssainy A. Rady, W. A. Hassanein, T. A. Elhaddad

**Affiliations:** 1 I.S.S.R, Cairo University, Giza, Egypt; 2 Faculty of Science, Tanta University, Tanta, Egypt

**Keywords:** Power Lomax distribution, Maximum likelihood, Bladder cancer, Hazard function, Goodness of fit

## Abstract

A three-parameters continuous distribution, namely, Power Lomax distribution (POLO) is proposed and studied for remission times of bladder cancer data. POLO distribution accommodate both inverted bathtub and decreasing hazard rate. Several statistical and reliability properties are derived. Point estimation via method of moments and maximum likelihood and the interval estimation are also studied. The simulation schemes are calculated to examine the bias and mean square error of the maximum likelihood parameter estimators. Finally, a real data application about the remission time of bladder cancer is used to illustrate the usefulness of the proposed distribution in modelling real data application. The characteristics of the fitting data using the proposed distribution are compared with known extensions of Lomax distribution. The comparison showed that the POLO distribution outfit most well-known extensions of Lomax distribution.

## Background

The Lomax (1954), or Pareto II, distribution introduced originally for modeling business failure data, moreover it has been widely applied in a variety of contexts. Hassan and Al-Ghamdi (2009) mentioned that it used for reliability modelling and life testing. The distribution has been used for modeling different data which studied by so many authors, Harris (1968) used Lomax distribution for income and wealth data, Atkinson and Harrison (1978) used it for modelling business failure data, while Corbelini et al. (2007) used it to model firm size and queuing problems. It has also found application in the biological sciences and even for modelling the distribution of the sizes of computer files on servers, Holland et al. (2006). Some authors, such as Bryson (1974), has suggested the use of this distribution as an alternative to the exponential distribution when the data are heavy-tailed.

A random variable *X* has the Lomax distribution with two parameters *α* and *λ* if it has cumulative distribution function (CDF) (for *x* > 0) given by

1 $$F\left( x \right) = 1 - \left( {1 + \frac{x}{\lambda }} \right)^{ - \alpha }$$

where, *α* > 0 and *λ* > 0 are the shape and scale parameters respectively. The probability density function (PDF) corresponding to (1) reduces to

2 $$f\left( x \right) = \frac{\alpha }{\lambda }\left( {1 + \frac{x}{\lambda }} \right)^{{ - \left( {\alpha + 1} \right)}} ,\quad x > 0,\alpha ,\lambda > 0$$

Lomax distribution can be motivated in a number of ways, e.g. Balkema and Haan (1974) showed that, it arises as the limit distribution of residual lifetime at old age, Dubey (1970) presented that it can be derived as a special case of a particular compound gamma distribution; and Tadikamalla (1980) relates Lomax distribution to Burr family. On the other hand, Lomax distribution is used as the basis for several generalizations. For example, Al-Awadhi and Ghitany (2001) use Lomax distribution as a mixing distribution for the Poisson parameter and derive a discrete Poisson-Lomax distribution; and Punathumparambath (2011) introduced the double-Lomax distribution and applied it to the IQ data. The record statistics of Lomax distribution has been studied by both Ahsanullah (1991) and Balakrishnan and Ahsanullah (1994). The implications of various forms of right-truncation and right-censoring are discussed by Myhre and Saunders (1982), Childs et al. (2001), Cramer and Schmiedt (2011) and others.

In the literature, some extensions of the Lomax distribution are available such as the Marshall–Olkin extended-Lomax (MOEL) by Ghitany et al. (2007) and Gupta et al. (2010), Beta–Lomax (BL), Kumaraswamy Lomax (KwL), McDonald-Lomax (McL) by Lemonte and Cordeiro (2013), Gamma-Lomax (GL) by Cordeiro et al. (2013) and Exponentiated Lomax (EL) by Abdul-Moniem (2012).

The McLomax density function (Lemonte and Cordeiro 2013) with five parameters $\alpha , \beta , a, \eta$ and *c*, denoted by McLomax $\left( {\alpha , \beta , a, \eta , c} \right),$ is expressed as

3 $$f\left( x \right) = \frac{{c\alpha \beta^{\alpha } \left( {\beta + x} \right)^{{ - \left( {\alpha + 1} \right)}} }}{{B\left( {ac^{ - 1} ,\eta + 1} \right)}}\left( {1 - \left( {\frac{\beta }{\beta + x}} \right)^{\alpha } } \right)^{a - 1} \left( {1 - \left( {1 - \left( {\frac{\beta }{\beta + x}} \right)^{\alpha } } \right)^{c} } \right)^{\eta } \quad x > 0$$

The CDF corresponding to Eq. (3) is given by

4 $$F\left( x \right) = I_{{\left\{ {1 - \beta^{\alpha } \left( {\beta + x} \right)^{ - \alpha } } \right\}^{c} }} \left( {ac^{ - 1} ,\eta + 1} \right)\quad x > 0$$

where, *I*_*y*_(*a*, *b*) is the incomplete Beta function.

Evidently, the density function (3) generalized several distributions as special sub-models not previously considered in the literature. In fact, Lomax distribution (with parameters *α* and *β*) is clearly a basic example for *a* = *c* = 1 and *η* = 0. BLomax and KwLomax distributions are new models which arise for *c* = 1 and *a* = *c*, respectively. For *η* = 0 and *c* = 1, it leads to a new distribution referred to as the ELomax distribution that extends the exponentiated standard Lomax (ESLomax) distribution for $\beta = 1$ Gupta et al. (1998).

The McLomax distribution can also be applied in engineering as the Lomax distribution. Arnold (1983) used this distribution to model reliability and survival problems. The McLomax distribution allows for greater flexibility of its tails and can be widely applied in many areas.

El-Bassiouny et al. (2015) introduced Exponential Lomax (Exp.Lomax) distribution with (CDF)

5 $$F\left( x \right) = 1 - e^{{ - \lambda \times \left( {\frac{\beta }{x + \beta }} \right)^{ - \alpha } }} ,\quad x \ge - \beta ,\alpha ,\beta ,\lambda > 0$$

Cordeiro et al. (2013) presented a three-parameters Gamma–Lomax (GL) distribution based on a versatile and flexible gamma generator proposed by Zografos and Balakrishnan (2009) using Stacy’s generalized gamma distribution and record value theory. The GL CDF is given by

6 $$F\left( x \right) = \frac{{\varGamma \left[ {a,\alpha {\text{Log}}\left[ {1 + \frac{x}{\beta }} \right]} \right]}}{\varGamma \left[ a \right]}, \quad x > 0,\alpha ,a,\beta > 0$$

where, α and a are shape parameters and β is a scale parameter.

Tahir et al. (2015) introduced the four parameters Weibull Lomax (WLomax) distribution with (CDF)

7 $$F\left( x \right) = 1 - {{e}}^{{\left( { - a\left( {\left( {1 + \left( {\frac{x}{\beta }} \right)} \right)^{\alpha } - 1} \right)^{b} } \right)}} \quad x > 0,a,b,\alpha ,\beta > 0$$

Al-Zahrania and Sagorb (2014) introduced Poisson-Lomax distribution (PLD) with CDF

8 $$F\left( x \right) = 1 - \frac{{1 - {{e}}^{{ - \lambda \left( {1 + \beta x} \right)^{ - \alpha } }} }}{{1 - {{e}}^{ - \lambda } }},\quad x > 0;\alpha ,\beta ,\lambda > 0$$

This distribution is a compound distribution of the zero truncated Poisson and Lomax distributions. The Extended Poisson-Lomax distribution (Ext.PLD) is introduced by Al-Zahrani (2015) with (CDF)

9 $$F\left( x \right) = 1 - \left( {1 + \beta x} \right)^{ - \alpha } {{e}}^{{ - \lambda \left( {1 - \left( {1 + \beta x} \right)^{ - \alpha } } \right)}},\quad x > 0;\lambda \ge 0,\alpha ,\beta > 0$$

Ashour and Eltehiwy (2013) proposed the transmuted exponentiated Lomax (TE-Lomax) distribution with (CDF)

10 $$F\left( x \right) = \left( {1 - \left( {1 + \gamma x} \right)^{ - \theta } } \right)^{\alpha } \left( {\left( {1 + \lambda } \right) - \lambda \left( {1 - \left( {1 + \gamma x} \right)^{ - \theta } } \right)^{\alpha } } \right)$$

where, $$x > 0;\lambda ,\gamma ,\theta ,\alpha > 0.$$

Using power transformation of a random variable may offer a more flexible distribution model by adding a new parameter. Ghitany et al. (2013) introduced two parameters distribution called power Lindley distribution and this model provides more flexibility than Lindley distribution.

The PDF of power Lindley distribution is given by

$$f\left( x \right) = \frac{{\alpha \beta^{2} }}{\beta + 1}\left( {1 + x^{\alpha } } \right)x^{\alpha - 1} e^{{ - \beta x^{\alpha } }} ,\quad x > 0,\alpha ,\beta > 0.$$

This paper is organized as follows; section “Model formulation” introduces the power Lomax (POLO) model formulation. The structural characteristics of POLO distribution including the behavior of the probability density function, the hazard rate function, the reversed hazard rate function, the (reversed) residual life, the entropy measures, the stress strength parameter, the moments and the associated moments, the order statistics and extreme values and finally the mean deviation and quantile function are studied in section “Structural characteristics”. Section “Methods of estimation” concerns with the point and interval estimations of POLO distribution. Simulation schemes are obtained in section “Simulation studies”. Finally, a real data life application of bladder cancer data are illustrated the potential of POLO distribution compared with other distributions in section “Application”.

## Model formulation

A new extension of the Lomax distribution is proposed by considering the power transformation $X = T^{{\frac{1}{\beta }}}$, where the random variable *T* follows Lomax distribution with parameters *α*, *λ*. The distribution of *X* is referred to as Power Lomax distribution. Symbolically, it is abbreviated by $X \sim POLO\left( {\alpha ,\beta ,\lambda } \right)$ to indicate that the random variable *X* has the power Lomax distribution with parameters *α*, *β* and *λ*.

The PDF of the Power Lomax distribution (POLO) is defined by

11 $$f\left( x \right) = \alpha \beta \lambda^{\alpha } x^{\beta - 1} \left( {\lambda + x^{\beta } } \right)^{ - \alpha - 1} ,\quad x > 0,\alpha ,\beta ,\lambda > 0.$$

The corresponding cumulative distribution function (CDF) of POLO distribution is given by

12 $$F\left( x \right) = 1 - \lambda^{\alpha } \left( {x^{\beta } + \lambda } \right)^{ - \alpha },\quad x > 0,\alpha ,\beta ,\lambda > 0.$$

The reliability (survival) function of POLO distribution is given by,

13 $$S\left( x \right) = 1 - F\left( x \right) = \lambda^{\alpha } \left( {x^{\beta } + \lambda } \right)^{ - \alpha } ,\quad x > 0,\alpha ,\beta ,\lambda > 0.$$

## Structural characteristics

In this section, we study the structural characteristics for POLO distribution. In particular, if $X \sim POLO\left( {\alpha ,\beta ,\lambda } \right)$ then the functional behavior of the density function and of the hazard function, reversed hazard function, mean residual life function and others are derived and studied in detail.

### Behavior of the probability density function of Power Lomax distribution

#### **Theorem 1**

*The PDF of Power Lomax distribution f(x) defined by Eq.* (11) *is*

- *Unimodal if* $$\alpha > 0,\beta > 1,\lambda > 0.$$
- *Decreasing if* $$\alpha > 0,0< {\beta \le 1,\lambda } > 0.$$

#### *Proof*

Since, $$\ln f\left( x \right) = {\text{Ln}}\left[ \alpha \right] + {\text{Ln}}\left[ \beta \right] + \alpha {\text{Ln}}\left[ \lambda \right] + \left( {\beta - 1} \right){\text{Ln}}\left[ x \right] - \left( {\alpha + 1} \right){\text{Ln}}\left[ {\lambda + x^{\beta } } \right].$$

It follows that,

$$\frac{d\ln f\left( x \right)}{dx} = \frac{ - 1 + \beta }{x} - \frac{{x^{ - 1 + \beta } \left( {1 + \alpha } \right)\beta }}{{x^{\beta } + \lambda }}.$$

For $0 < \beta \le 1$, $\frac{d\ln f\left( x \right)}{dx} < 0$, then $f\left( x \right)$ is decreasing. For *β* > 1, $\frac{d\ln f\left( x \right)}{dx} = 0$ implies that *f*(*x*) has a mode at *x*_0_, where

$$x_{0} = \left( {\frac{{\left( { - 1 + \beta } \right)\lambda }}{1 + \alpha \beta }} \right)^{{\frac{1}{\beta }}} .$$

Since, at *α* > 0, *β* > 1, *λ* > 0

$$\frac{{{\text{d}}^{2} \ln {\text{f}}\left( x \right)}}{{{\text{d}}x^{2} }} = \frac{{x^{2\beta } \left( {1 + \alpha \beta } \right) - x^{\beta } \left( { - 1 + \beta } \right)\left( {2 + \beta + \alpha \beta } \right)\lambda - \left( { - 1 + \beta } \right)\lambda^{2} }}{{x^{2} \left( {x^{\beta } + \lambda } \right)^{2} }}.$$

Then $$\frac{{ {\text{d}}^{2} \ln {\text{f}}\left( {x_{0} } \right)}}{{{\text{d}}x^{2} }} = - \frac{{\left( {\beta - 1} \right)\left( {1 + \alpha \beta } \right)}}{1 + \alpha }\left( {\frac{{\left( {\beta - 1} \right)\lambda }}{1 + \alpha \beta }} \right)^{ - 2/\beta } < 0.$$

**Fig. 1:**
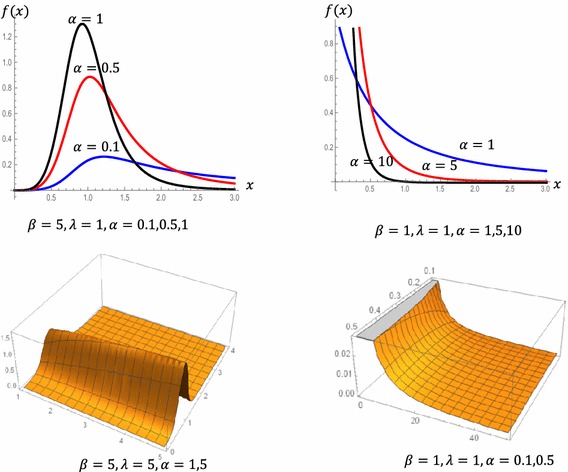
PDF of the POLO distribution

 Figure 1 is the plots of the POLO density function for different values of *α*, *β* and *λ*.

### Hazard rate function

The survival function examines the chance of breakdowns of organisms or technical units etc. occur beyond a given point in time. To monitor the lifetime of a unit across the support of its lifetime distribution, the hazard rate is used. The hazard rate (HRF) measures the tendency to fail or to die depending on the age reached and it thus plays a key role in classifying lifetime distributions. Generally, hazard rates are monotonic (increasing or decreasing) or non-monotonic (bathtub or inverted bathtub) functions, Rinne (2014).

From Eqs. (11), (13), the hazard rate function (HRF) of the power Lomax is defined by

14 $$h\left( x \right) = \frac{{x^{\beta - 1} \alpha \beta }}{{x^{\beta } + \lambda }}, \quad x > 0,\alpha ,\beta ,\lambda > 0.$$

The following theorem gives conditions under which the HRF, given by (14), is a decreasing hazard rate (DHR) and upside down bathtub (inverted bathtub IBT) also named by (IDHR Increasing–Decreasing Hazard Rate).

#### **Theorem 2**

*The hazard rate function of power Lomax distribution* $\left( {\alpha ,\beta ,\lambda } \right)$ *defined by Eq.* (14) *is*

- *IBT if* $$\alpha > 0,\beta > 1,\lambda > 0$$
- *DHR if* $$\alpha > 0,0< {\beta \le 1,\lambda } > 0$$

#### *Proof*

Since,

$$h^{\prime}\left( x \right) = - \frac{{x^{ - 2 + \beta } \alpha \beta \left( {x^{\beta } + \lambda - \beta \lambda } \right)}}{{\left( {x^{\beta } + \lambda } \right)^{2} }}.$$

For $0 < \beta \le 1$, $h^{\prime}\left( x \right) < 0$, then *h*(*x*) is decreasing.

For $\alpha > 0,\beta > 1,\lambda > 0$, $h^{\prime}\left( x \right) = 0$ implies that *h*(*x*) has a global maximum at

$$x_{1} = \left( {\lambda \beta - \lambda } \right)^{{\frac{1}{\beta }}}$$

Therefore, *h*(*x*) is inverted bathtub shaped (IBT)

where, $$h^{{\prime \prime }} \left( x \right)<{0 at x_{1} , \alpha }> 0,\beta > 1,\lambda > 0$$

$$h^{{\prime \prime }} \left( {x_{1} } \right) = \frac{{\alpha \beta \left( {\beta - 1} \right)^{{\frac{2\beta - 3}{\beta }}} \left( {8 - 6\beta - \beta^{2} } \right)\left( \lambda \right)^{ - 3/\beta } }}{{\left( {\beta - 2} \right)^{3} }} < 0.$$

**Fig. 2:**
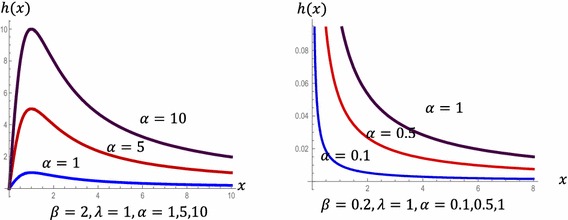
HRF of the POLO distribution

**Fig. 3:**
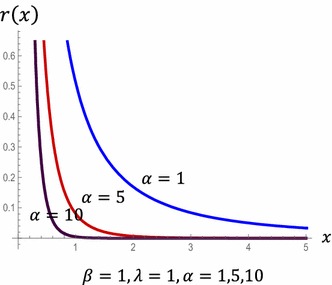
Reversed hazard function of the POLO distribution

HRF of the POLO distribution are displayed in Fig. 2 for different values of *α*, *β* and *λ* (Fig. 3).

### Reversed hazard rate

The reversed hazard rate can be defined as the conditional random variable [*t* − *X*/*X* ≤ *t*] which denotes the time elapsed from the failure of a component given that its life is less than or equal to *t*. This random variable is called also the inactivity time or time since failure.

Using Eqs. (11), (12), the reversed hazard function of the POLO distribution can be given by

$$r\left( x \right) = \frac{f\left( x \right)}{F\left( x \right)}.$$

Thus $$r\left( x \right) = \frac{{\left( {\alpha \beta \lambda^{\alpha } x^{\beta - 1} \left( {\lambda + x^{\beta } } \right)^{ - \alpha - 1} } \right)}}{{1 - \lambda^{\alpha } \left( {x^{\beta } + \lambda } \right)^{ - \alpha } }},\quad x > 0,\alpha ,\beta ,\lambda > 0.$$

### (Reversed) Residual life functions

Residual life and reversed residual life random variables are used extensively in risk analysis. Accordingly, we investigate some related statistical functions, such as survival function, mean and variance in connection with POLO distribution. The residual life is described by the conditional random variable $R_{(t)} = X - t | X > t, \quad t \ge 0$, and defined as the period from time *t* until the time of failure. Analogously, the reversed residual life can be defined as $\bar{R}_{\left( t \right)} = t - X|X \le t$ which denotes the time elapsed from the failure of a component given that its life ≤ *t*.

i. Residual lifetime function

The survival function of the residual lifetime $S_{\left( t \right)},\, t \ge 0$, for POLO distribution is given by

$$S_{{R_{\left( t \right)} }} \left( x \right) = \frac{{S\left( {x + t} \right)}}{S\left( t \right)} = \left( {t^{\beta } + \lambda } \right)^{\alpha } \left( {\left( {t + x} \right)^{\beta } + \lambda } \right)^{ - \alpha } ,\quad x > 0.$$

and its PDF is

$$f_{{R_{\left( t \right)} }} \left( x \right) = \left( {t + x} \right)^{ - 1 + \beta } \alpha \beta \left( {t^{\beta } + \lambda } \right)^{\alpha } \left( {\left( {t + x} \right)^{\beta } + \lambda } \right)^{ - 1 - \alpha }$$

Consequently, the hazard rate function of *R*_(*t*)_ has the following form

$$h_{{R_{\left( t \right)} }} \left( x \right) = \frac{{\left( {t + x} \right)^{ - 1 + \beta } \alpha \beta }}{{\left( {t + x} \right)^{\beta } + \lambda }} .$$

ii. Mean residual life function

The mean residual life (MRL) function $MRL = E(X - x\left| {{\text{X}} > {\text{x}}} \right.)$ of power Lomax distribution is given by

$$MRL = \frac{1}{S\left( x \right)} \int\nolimits_{x}^{\infty } S\left( t \right){\text{d}}t.$$

Thus $$MRL = \frac{{\left( { - \lambda } \right)^{{ - \alpha + \frac{1}{\beta }}} \left( {x^{\beta } + \lambda } \right)^{\alpha } {\text{Beta}}\left[ { - x^{ - \beta } \lambda ,\alpha - \frac{1}{\beta },1 - \alpha } \right]}}{\beta } , x > 0,\alpha ,\beta ,\lambda > 0$$.

#### **Theorem 3**

*The behavior of the MRL for POLO distribution is*

1. *MRL is increasing for* $\alpha > 0,0<{\beta \le 1,\lambda }> 0.$
2. *MRL is bathtub (BT) for* $\alpha > 0,\beta > 1,\lambda > 0.$

#### *Proof*

Finkelstein (2002) proved that when the hazard rate function is monotonically increasing (decreasing), then the corresponding MRL function will be monotonically decreasing (increasing). The sufficient conditions for MRL to be IBT (BT) is that hazard rate function has BT (IBT) shapes and $f\left( 0 \right)\mu_{1} \left( 0 \right) > 1 \left( { \le 1} \right)$; where *μ*_1_(0) is MRL at *x* = 0 Gupta et al. (1999). Hence, $f\left( 0 \right)\mu_{1} \left( 0 \right) < 1$ and the HRF is IBT, then the MRL is BT at $\alpha > 0,\beta > 1,\lambda > 0$. Moreover, MRL is increasing since HRT is decreasing at $\alpha > 0,0 <{\beta \le 1,\lambda }> 0$.

**Fig. 4:**
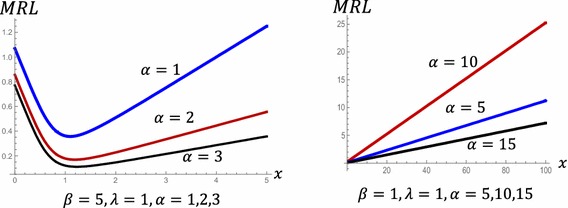
MRL for POLO distribution

Figure 4 displays the behavior of MRL of POLO distribution at different values of the parameters

iii. Reversed residual life function

The survival function of the reversed residual lifetime $\bar{R}_{\left( t \right)}$ for POLO distribution is given by

$$S_{{\bar{R}\left( t \right)}} \left( x \right) = \frac{{F\left( {t - x} \right)}}{F\left( t \right)} = \frac{{1 - \lambda^{\alpha } \left( {\left( {t - x} \right)^{\beta } + \lambda } \right)^{ - \alpha } }}{{1 - \lambda^{\alpha } \left( {t^{\beta } + \lambda } \right)^{ - \alpha } }} ,\quad 0 \le x < t.$$

hence the probability density function of $\bar{R}_{\left( t \right)}$ takes the following form

$$f_{{_{{\bar{R}\left( t \right)}} }} \left( x \right) = - \frac{{\left( {t - x} \right)^{ - 1 + \beta } \alpha \beta \lambda^{\alpha } \left( {\left( {t - x} \right)^{\beta } + \lambda } \right)^{ - 1 - \alpha } }}{{1 - \lambda^{\alpha } \left( {t^{\beta } + \lambda } \right)^{ - \alpha } }} .$$

Consequently the hazard rate function of the reversed residual lifetime $\bar{R}_{\left( t \right)}$ has the following form

$$h_{{_{{\bar{R}\left( t \right)}} }} \left( x \right) = \frac{{\left( {t - x} \right)^{ - 1 + \beta } \alpha \beta \lambda^{\alpha } }}{{\left( {\left( {t - x} \right)^{\beta } + \lambda } \right)\left( {\lambda^{\alpha } - \left( {\left( {t - x} \right)^{\beta } + \lambda } \right)^{\alpha } } \right)}} .$$

### Moments and associated measures

The *r*th raw moments (about the origin) of power Lomax distribution is given by

$$\mu_{r}^{{\prime }} = \int \nolimits_{0}^{\infty } x^{r} \alpha \beta \lambda^{\alpha } x^{\beta - 1} \left( {\lambda + x^{\beta } } \right)^{ - \alpha - 1} dx.$$

$$\mu_{r}^{\prime} = \frac{{\alpha \lambda^{r/\beta } \varGamma \left[ {\alpha - \frac{r}{\beta }} \right]\varGamma \left[ {\frac{r + \beta }{\beta }} \right]}}{{\varGamma \left[ {1 + \alpha } \right]}}.$$

The first four moments about the origin of the power Lomax distribution have been obtained as follows

$$\mu_{1}^{\prime} = \frac{{\alpha \lambda^{{\frac{1}{\beta }}} \varGamma \left[ {\alpha - \frac{1}{\beta }} \right]\varGamma \left[ {\frac{1}{\beta }} \right]}}{{\beta \varGamma \left[ {1 + \alpha } \right]}}\quad \quad \mu_{2}^{\prime} = \frac{{\alpha \lambda^{2/\beta } \varGamma \left[ {\alpha - \frac{2}{\beta }} \right]\varGamma \left[ {\frac{2 + \beta }{\beta }} \right]}}{{\varGamma \left[ {1 + \alpha } \right]}}.$$

$$\mu_{3}^{\prime} = \frac{{\alpha \lambda^{3/\beta } \varGamma \left[ {\alpha - \frac{3}{\beta }} \right] \varGamma \left[ {\frac{3 + \beta }{\beta }} \right]}}{{\varGamma \left[ {1 + \alpha } \right]}}\quad \quad \mu_{4}^{\prime} = \frac{{\alpha \lambda^{4/\beta } \varGamma \left[ {\alpha - \frac{4}{\beta }} \right]\varGamma \left[ {\frac{4 + \beta }{\beta }} \right]}}{{\varGamma \left[ {1 + \alpha } \right]}}.$$

Therefore, the mean and variance of power Lomax distribution respectively, are as follows

$$\mu = \frac{{\alpha \lambda^{{\frac{1}{\beta }}} \varGamma \left[ {\alpha - \frac{1}{\beta }} \right]\varGamma \left[ {\frac{1}{\beta }} \right]}}{{\beta \varGamma \left[ {1 + \alpha } \right]}},\quad \sigma^{2} = \frac{{\lambda^{{\frac{2}{\beta }}} \left( { - \varGamma \left[ {\alpha - \frac{1}{\beta }} \right]^{2} \varGamma \left[ {1 + \frac{1}{\beta }} \right]^{2} + \varGamma \left[ \alpha \right]\varGamma \left[ {\alpha - \frac{2}{\beta }} \right] \varGamma \left[ {\frac{2 + \beta }{\beta }} \right] } \right)}}{{\varGamma \left[ \alpha \right] ^{2} }}.$$

The first four central moments about the mean are then given as follows

$$\mu_{k} = E\left[ {\left( {x - \mu } \right)^{k} } \right].\quad \mu_{2} = \frac{{\alpha \lambda^{{\frac{2}{\beta }}} \left( { - \varGamma \left[ {\alpha - \frac{1}{\beta }} \right]^{2} \varGamma \left[ {\frac{1}{\beta }} \right]^{2} + 2\beta \varGamma \left[ \alpha \right]\varGamma \left[ {\alpha - \frac{2}{\beta }} \right]\varGamma \left[ {\frac{2}{\beta }} \right]} \right)}}{{\beta^{2} \varGamma \left[ \alpha \right]\varGamma \left[ {1 + \alpha } \right]}}.$$

$$\mu_{3} = \frac{1}{{\beta^{3} \varGamma \left[ \alpha \right]^{3} }}\lambda^{{\frac{3}{\beta }}} \left( {2\varGamma \left[ {\alpha - \frac{1}{\beta }} \right]^{3} \varGamma \left[ {\frac{1}{\beta }} \right]^{3} - 6\beta \varGamma \left[ \alpha \right]\varGamma \left[ {\alpha - \frac{2}{\beta }} \right]\varGamma \left[ {\alpha - \frac{1}{\beta }} \right]\varGamma \left[ {\frac{1}{\beta }} \right]\varGamma \left[ {\frac{2}{\beta }} \right] +\,3\beta^{2} \varGamma \left[ \alpha \right]^{2} \varGamma \left[ {\alpha - \frac{3}{\beta }} \right]\varGamma \left[ {\frac{3}{\beta }} \right]} \right).$$

$$\mu_{4} = \frac{1}{{\beta^{4} \varGamma \left[ \alpha \right]^{4} }}\lambda^{{\frac{4}{\beta }}} \left( { - 3\varGamma \left[ {\alpha - \frac{1}{\beta }} \right]\varGamma \left[ {\frac{1}{\beta }} \right]\left( {\varGamma \left[ {\alpha - \frac{1}{\beta }} \right]^{3} \varGamma \left[ {\frac{1}{\beta }} \right]^{3} - 4\beta \varGamma \left[ \alpha \right]\varGamma \left[ {\alpha - \frac{2}{\beta }} \right]\varGamma \left[ {\alpha - \frac{1}{\beta }} \right]\varGamma \left[ {\frac{1}{\beta }} \right]\varGamma \left[ {\frac{2}{\beta }} \right] + 4\beta^{2} \varGamma \left[ \alpha \right]^{2} \varGamma \left[ {\alpha - \frac{3}{\beta }} \right]\varGamma \left[ {\frac{3}{\beta }} \right]} \right) + \beta^{4} \varGamma \left[ \alpha \right]^{3} \varGamma \left[ {\alpha - \frac{4}{\beta }} \right]\varGamma \left[ {\frac{4 + \beta }{\beta }} \right]} \right).$$

The skewness and kurtosis measures can be obtained from the expressions respectively

$$\beta_{1} = - \left( {\frac{{\left( {2\varGamma \left[ {\alpha - \frac{1}{\beta }} \right]^{3} \varGamma \left[ {\frac{1}{\beta }} \right]^{3} - 6\beta \varGamma \left[ \alpha \right]\varGamma \left[ {\alpha - \frac{2}{\beta }} \right]\varGamma \left[ {\alpha - \frac{1}{\beta }} \right]\varGamma \left[ {\frac{1}{\beta }} \right]\varGamma \left[ {\frac{2}{\beta }} \right] + 3\beta^{2} \varGamma \left[ \alpha \right]^{2} \varGamma \left[ {\alpha - \frac{3}{\beta }} \right]\varGamma \left[ {\frac{3}{\beta }} \right]} \right)^{2} }}{{\left( {\varGamma \left[ {\alpha - \frac{1}{\beta }} \right]^{2} \varGamma \left[ {\frac{1}{\beta }} \right]^{2} - 2\beta \varGamma \left[ \alpha \right]\varGamma \left[ {\alpha - \frac{2}{\beta }} \right]\varGamma \left[ {\frac{2}{\beta }} \right]} \right)^{3} }}} \right).$$

$${ \beta_{2} = \left( - 3\varGamma \left[ {\alpha- \frac{1}{\beta }}\right]\varGamma \left[ {\frac{1}{\beta }}\right] \left(\varGamma\left[ {\alpha - \frac{1}{\beta }}\right]^{3} \varGamma\left[{\frac{1}{\beta }} \right]^{3} - 4\beta\varGamma \left[\alpha\right]\varGamma \left[ {\alpha -\frac{2}{\beta }}\right]\varGamma\left[ {\alpha - \frac{1}{\beta }}\right]\varGamma\left[{\frac{1}{\beta }} \right]\varGamma \left[{\frac{2}{\beta }}\right] + 4\beta^{2}\varGamma \left[ \alpha\right]^{2} \varGamma\left[ {\alpha -\frac{3}{\beta }}\right]\varGamma \left[{\frac{3}{\beta }} \right]\right) +\beta^{4} \varGamma \left[\alpha \right]^{3} \varGamma\left[{\alpha - \frac{4}{\beta }}\right]\varGamma \left[ {\frac{4 +\beta}{\beta }} \right] \right)\bigg/ {{\beta^{4}\left( {\varGamma \left[ {\alpha -\frac{1}{\beta }} \right]^{2}\varGamma \left[ {1 + \frac{1}{\beta}} \right]^{2} - \varGamma\left[ \alpha \right]\varGamma \left[{\alpha - \frac{2}{\beta }}\right] \varGamma \left[ {\frac{2 +\beta }{\beta }} \right]}\right)^{2} }}}.$$

### Order statistics and extreme values

The distribution of extreme values plays an important role in statistical applications. In this section the probability and cumulative function of order statistics are introduced and the limiting distribution of minimum and the maximum arising from the power Lomax distribution can then be derived.

#### Probability and cumulative function of order statistics

Suppose $X_{1} ,X_{2} , \ldots \ldots .,X_{n}$ is a random sample from power Lomax distribution. Let $X_{1:n} < X_{2:n} < \cdots < X_{n:n}$ denote the corresponding order statistics. The probability density function and the cumulative distribution function of the *k*th order statistic of POLO distribution, say $Y = X_{j:n}$ are given by

$$\begin{aligned} f_{Y} \left( y \right) & = \frac{n!}{{\left( {k - 1} \right)!\left( {n - k} \right)!}}F^{k - 1} \left( y \right)\left\{ {1 - F\left( y \right)} \right\}^{n - k} f\left( y \right). \\ & = \frac{n!}{{\varGamma \left[ k \right]\varGamma \left[ {1 - k + n} \right]}} \cdot \left[ {\alpha \beta \lambda^{\alpha } y^{\beta - 1} \left( {\lambda + y^{\beta } } \right)^{ - \alpha - 1} } \right] \cdot \left[ {1 - \lambda^{\alpha } \left( {y^{\beta } + \lambda } \right)^{ - \alpha } } \right]^{k - 1} \cdot \left[ {\lambda^{\alpha } \left( {y^{\beta } + \lambda } \right)^{ - \alpha } } \right]^{n - k} . \\ \end{aligned}$$

Moreover,

$$\begin{aligned} F_{Y} \left( y \right) & = \mathop \sum \limits_{m = k}^{n} \left( {\begin{array}{*{20}c} n \\ m \\ \end{array} } \right)F^{m} \left( y \right) \times \left[ {1 - F\left( y \right)} \right]^{n - m} . \\ F_{Y} \left( y \right) & = \mathop \sum \limits_{m = k}^{n} \left( {\begin{array}{*{20}c} n \\ m \\ \end{array} } \right)\left( {1 - \lambda^{\alpha } \left( {y^{\beta } + \lambda } \right)^{ - \alpha } } \right)^{m} \times \left[ {\lambda^{\alpha } \left( {y^{\beta } + \lambda } \right)^{ - \alpha } } \right]^{n - m} . \\ \end{aligned}$$

#### Limiting distributions of extreme values

Let $m_{n} = X_{1:n} = { \hbox{min} }\left[ {X_{1} , {\text{X}}_{2} , . . . ,{\text{X}}_{n} } \right]$ and $M_{n} = X_{n:n} = { \hbox{max} }\left[ {X_{1} , {\text{X}}_{2} , . . . ,{\text{X}}_{n} } \right]$ arising from Power Lomax distribution. The limiting distributions of $X_{1:n}$ and $X_{n:n}$ can be obtained by the following theorem.

##### **Theorem 4**

*Let m*_*n*_ *and M*_*n*_ *be the minimum and the maximum of a random sample from the Power Lomax distribution, respectively. Then*

1. $$\mathop {\lim }\nolimits_{n \to \infty } p\left( {\frac{{m_{n} - a_{n} }}{{b_{n} }} \le x} \right) = 1 - { \exp }\left( { - x^{\beta } } \right);\quad x > 0.$$
2. $$\mathop {\lim }\nolimits_{n \to \infty } p\left( {\frac{{M_{n} - c_{n} }}{{d_{n} }} \le x} \right) = { \exp }\left( { - x^{ - \alpha \beta } } \right);\quad x > 0.$$

*where; a*_*n*_ = 0, $$b_{n} = \frac{1}{{F^{ - 1} \left( {\frac{1}{n}} \right)}}, c_{n} = 0$$ *and* $$d_{n} = \frac{1}{{F^{ - 1} \left( {1 - \frac{1}{n}} \right)}}$$.

##### *Proof*

1. Using L’Hospital rule, we have
  $$\mathop {\lim }\limits_{{\varepsilon \to 0^{ + } }} \frac{{F\left( {F^{ - 1} \left( 0 \right) + \varepsilon x} \right)}}{{F\left( {F^{ - 1} \left( 0 \right) + \varepsilon } \right)}} = \mathop {\lim }\limits_{{\varepsilon \to 0^{ + } }} \frac{{F\left( {\varepsilon x} \right)}}{F\left( \varepsilon \right)} = \mathop {\lim }\limits_{{\varepsilon \to 0^{ + } }} \frac{{xf\left( {\varepsilon x} \right)}}{f\left( \varepsilon \right)} = x^{\beta } .$$

Therefore by Theorem (8.3.6) of Arnold et al. (1992), the minimal domain of attraction of the Power Lomax distribution is the Weibull distribution, and thus (i) is proved.

2. Using L’Hospital rule, we have
  $$\mathop {\lim }\limits_{t \to \infty } \frac{{1 - F\left( {tx} \right)}}{1 - F\left( t \right)} = \mathop {\lim }\limits_{t \to \infty } \frac{{xf\left( {tx} \right)}}{f\left( t \right)} = x^{ - \alpha \beta } .$$

Therefore, by Theorem (1.6.2) and Corollary (1.6.3) in Leadbetter et al. (1987), the maximal domain of attraction of the Power Lomax distribution is Fréchet distribution, and thus (ii) is proved.

### Quantiles and mean deviation

Quantiles are useful measures because they are less susceptible to long-tailed distributions. Also, quantiles may be more useful descriptive statistics than means and other moment-related statistics.

Let *X* denotes a random variable with the POLO probability density function, the quantile function, *Q*(*p*) is given by

$$Q\left( p \right) = \inf \left\{ {x\varepsilon R:F\left( x \right) \ge p} \right\},\quad where\, 0 < p < 1.$$

By inverting the cumulative distribution function, the quantile function for POLO distribution has the following form

$$Q\left( p \right) = \lambda^{{\frac{1}{\beta }}} \left( {\left( {1 - p} \right)^{{ - \frac{1}{\alpha }}} - 1} \right)^{{\frac{1}{\beta }}} .$$

Consequently, the first, median and the third quartiles of the power Lomax distribution are respectively given by

$$Q_{1} = F^{ - 1} \left( {\frac{1}{4}} \right) = \lambda^{{\frac{1}{\beta }}} \left( {\left( {\frac{3}{4}} \right)^{{ - \frac{1}{\alpha }}} - 1} \right)^{{\frac{1}{\beta }}} ,\quad Q_{2} = F^{ - 1} \left( {\frac{1}{2}} \right) = \lambda^{{\frac{1}{\beta }}} \left( {\left( {\frac{1}{2}} \right)^{{ - \frac{1}{\alpha }}} - 1} \right)^{{\frac{1}{\beta }}}$$

In statistics, the mean deviation about the mean and mean deviation about the median measure the amount of scatter in a population. For a random variable *X* with PDF, *f* (*x*), distribution function *F*(*x*), mean *μ* = *E*(*X*) and *M* = Median(*X*), mean deviation about the mean and mean deviation about the median are defined by $\eta_{1} \left( x \right) = \int_{0}^{\infty } {\left| {x - \mu } \right|f\left( x \right)dx}$ and $\eta_{2} \left( x \right) = \int_{0}^{\infty } {\left| {x - M} \right|f\left( x \right)dx}$ respectively.

The next theorem gives such mean deviation for POLO random variable.

#### **Theorem 5**

*If X is POLO random variable, then*

$$\eta_{1} \left( x \right) = 2\mu F\left( \mu \right) - 2\mu + 2\alpha \left( { - \lambda } \right)^{{ - \alpha + \frac{1}{\beta }}} \lambda^{\alpha } {\text{Beta}}\left[ { - \lambda \mu^{ - \beta } ,\alpha - \frac{1}{\beta }, - \alpha } \right].$$

*and*

$$\eta_{2} \left( x \right) = 2MF\left( M \right) - M + 2\alpha \left( { - \lambda } \right)^{{ - \alpha + \frac{1}{\beta }}} \lambda^{\alpha } {\text{Beta}}\left[ { - M^{ - \beta } \lambda ,\alpha - \frac{1}{\beta }, - \alpha } \right].$$

*where F(.) is the CDF of POLO distribution, given by Eq.* *(* 12 *) and μ,* *M are the mean and median of this distribution, respectively, given by* $\mu = \frac{{\alpha \lambda^{{\frac{1}{\beta }}} \varGamma \left[ {\alpha - \frac{1}{\beta }} \right]\varGamma \left[ {\frac{1}{\beta }} \right]}}{{\beta \varGamma \left[ {1 + \alpha } \right]}}$ and $M = \lambda^{{\frac{1}{\beta }}} \left( {\left( {\frac{1}{2}} \right)^{{ - \frac{1}{\alpha }}} - 1} \right)^{{\frac{1}{\beta }}}$.

#### *Proof*

From the definitions of *η*_1_(*x*) and *η*_2_(*x*), we can show that

$$\eta_{1} \left( x \right) = 2\mu F\left( \mu \right) - 2\mu + 2\int \nolimits_{\mu }^{\infty } xf\left( x \right).$$

and

$$\eta_{2} \left( x \right) = 2MF\left( M \right) - M + 2\int \nolimits_{M}^{\infty } xf\left( x \right).$$

which complete the proof.

### Shannon’s & Rényi and Song’s entropy measures

Entropy is a measure of randomness, disorder, chaos or loss of information of systems. It can be used in many essential fields such as chemistry, physics and biology as a driving force for protein unfolding or catalysis of enzymes.

(i) For a continuous random variable X with density function *f*(*x*), Shannon’s entropy is defined by

$$S_{H} = - \int \nolimits_{0}^{\infty } f\left( x \right)logf\left( x \right)dx.$$

Shannon’s entropy for POLO distribution is defined by

$$S_{H} = - \int\nolimits_{0}^{\infty } f\left( x \right)logf\left( x \right)dx.$$

$$S_{H} = - \alpha \beta \lambda^{\alpha }\int \nolimits_{0}^{\infty } x^{\beta - 1} \left( {\lambda + x^{\beta } } \right)^{ - \alpha - 1} {\text{Log}}\left[ {\alpha \beta \lambda^{\alpha } x^{\beta - 1} \left( {\lambda + x^{\beta } } \right)^{ - \alpha - 1} } \right]{\text{d}}x.$$

$$S_{H} = - \alpha \beta \lambda^{\alpha } \left( {\frac{{\left( { - 1 + \beta } \right)\lambda^{ - \alpha } \left( { - {\text{HarmonicNumber}}\left[ { - 1 + \alpha } \right] + {\text{Log}}\left[ \lambda \right]} \right)}}{{\alpha \beta^{2} }} - \frac{{\left( {1 + \alpha } \right)\lambda^{ - \alpha } \left( {1 + \alpha {\text{Log}}\left[ \lambda \right]} \right)}}{{\alpha^{2} \beta }} + \frac{{\lambda^{ - \alpha } {\text{Log}}\left[ {\alpha \beta \lambda } \right]}}{\beta }} \right).$$

$$S_{H} = 1 + \frac{1}{\alpha } + \alpha {\text{Log}}\left[ \lambda \right] - \alpha {\text{Log}}\left[ {\alpha \beta \lambda } \right] + \frac{{{\text{Log}}\left[ \lambda \right] + \left( {\beta - 1} \right)\left( {{\text{EulerGamma}} + {\text{PolyGamma}}\left[ {0,\alpha } \right]} \right)}}{\beta }.$$

Some numerical values for Shannon’s entropy are given in Table 1. It’s seems that the entropy decreases with increasing $\alpha , \beta$, while decreases with increasing *λ*.

**Table 1:** Entropy for several arbitrary parameter values

Parameters	*λ* = 2, *β* = 0.2		*α* = 0.3, *β* = 0.5		*α* = 1.5, *λ* = 3
*α*↓	Entropy	*λ*↑	Entropy	*β*↓	Entropy
0.1	54.2431	0.2	4.6089	0.1	9.97512
0.5	13.1622	0.5	6.44148	0.4	4.25888
1	7.07517	1	7.82778	0.8	2.61302
1.5	4.48354	1.5	8.63871	1	2.15708
3	0.331546	3	10.025	1.3	1.65163
3.5	−1.12738	5.8	11.3435	2	0.87491
5	−3.6676	7	11.7196	3.5	−0.06843

(ii) Rényi entropy

Rényi entropy and Song’s measure are used to measure the intrinsic shape of the distribution.

Rényi entropy is defined by

$$I_{R} \left( \gamma \right) = \left( {1 - \gamma } \right)^{ - 1} \log \left( {\int_{R} {f^{\gamma } \left( x \right)dx) ,\quad \gamma > 0,\gamma \ne 1} } \right).$$

For POLO distribution, Rényi entropy is given by

$$\begin{aligned} I_{R} \left( \gamma \right) & = \left( {1 - \gamma } \right)^{ - 1} \log \left(\int\nolimits_{0}^{\infty } \left( {\alpha \beta \lambda^{\alpha } x^{\beta - 1} \left( {\lambda + x^{\beta } } \right)^{ - \alpha - 1} } \right)^{\gamma } \right)dx ,\quad \gamma > 0, \quad \gamma \ne 1 \\ & = \left( {1 - \gamma } \right)^{ - 1} {\text{Log}}\left[ {\left( {\alpha \beta \lambda^{\alpha } } \right)^{\gamma } \frac{{\lambda^{{ - \frac{ - 1 + \gamma + \alpha \beta \gamma }{\beta }}} \varGamma \left[ {\frac{{1 + \left( { - 1 + \beta } \right)\gamma }}{\beta }\left] \varGamma \right[\frac{ - 1 + \gamma + \alpha \beta \gamma }{\beta }} \right]}}{{\beta \varGamma \left[ {\gamma + \alpha \gamma } \right]}}} \right]. \\ \end{aligned}$$

(iii) Song’s measure of a distribution is defined by

$$S_{f} = \mathop {\lim }\limits_{\gamma \to 1} - 2\frac{{d I_{R} \left( \gamma \right)}}{d\gamma }$$

Hence, for POLO distribution:

$$\begin{aligned} \,\frac{{dI_{R} \left( \gamma\right)}}{d\gamma} &= \frac{1}{{\beta \left( {\gamma - 1} \right)^{2} }}\left(\left( {\gamma - 1} \right)\left( { - \beta {\text{Log}}\left[ {\alpha \beta \lambda^{\alpha } } \right] + {\text{Log}}\left[ \lambda \right] + \alpha \beta {\text{Log}}\left[\lambda \right]} \right)\right. \\ & \quad+ \beta{\text{Log}}\left[ \frac{{\left( {\alpha \beta \lambda^{\alpha } } \right)^{\gamma } \lambda^{{ -\frac{\gamma - 1 + \alpha \beta \gamma }{\beta }}} \varGamma \left[\frac{1 + \left( { - 1 + \beta } \right)\gamma }{\beta }\right] \varGamma \left[\frac{\gamma - 1 + \alpha \beta \gamma }{\beta } \right]}}{{\beta {\text{Gamma}}\left[ {\gamma + \alpha \gamma } \right]}}\right]+ \left( {1 + \alpha } \right)\beta \left( {\gamma - 1} \right){\text{PolyGamma}} [0,\gamma + \alpha \gamma] \\ & \quad \left. + ( - 1 + \beta + \gamma - \beta \gamma ){\text{PolyGamma}}\left[0,\frac{{1 + \left( { - 1 + \beta } \right)\gamma }}{\beta }\right] { - \left( {1 + \alpha \beta } \right)\left( { - 1 + \gamma } \right){\text{PolyGamma}}} \left[0,\frac{\gamma - 1 + \alpha \beta \gamma }{\beta }\right]\right). \\ \end{aligned}$$

By L’Hôpital’s rule, Song’s measure for POLO distribution is obtained as

$$S_{f} = \frac{1}{3}\pi^{2} \left( { - 1 + \beta } \right)^{2} - \left( {1 + \alpha \beta } \right)^{2} {\text{PolyGamma}}\left[ {1,\alpha } \right] - 2\left( {1 + \alpha } \right)^{2} \beta^{2} {\text{PolyGamma}}\left[ {1,1 + \alpha } \right]).$$

### Stress strength parameter

In lifetime models, the stress strength parameter, $R = P(X < Y)$, describes the lifetime component which has a random stress *X* that is subjected to a random strength *Y*. It plays a vital role in reliability. The component fails at the moment that the stress applied to it exceeds the strength, and the component will function satisfactorily whenever *X* < *Y*. The next theorem gives the stress-strength parameter for POLO distribution.

#### **Theorem 6**

*Let X and Y be two independent random variables distributed as POLO (α*_*1*_, *β*_*1*_, *λ*_*1*_ *) and POLO (α*_*2*_, *β*_*2*_, *λ*_*2*_ *) respectively, Then the stress strength parameter R is given as follows*

$$\begin{aligned} & R = \alpha_{1} \beta_{1} \lambda_{1}^{{\alpha_{1} }} \left[ {1 - \lambda_{2}^{{ - \beta_{2} \left( {\alpha_{2} + 1} \right)}} \lambda_{1}^{{\alpha_{1} \beta_{1} }} } \right]\left[ {\mathop \sum \limits_{j = 0}^{\infty } \left( {\begin{array}{*{20}c} { - \left( {\alpha_{2} + 1} \right)} \\ j \\ \end{array} } \right)\lambda_{2}^{{ - \beta_{2} j}} \frac{{{\text{Hypergeometric}}2{\text{F}}1\left[ {\alpha_{1} ,\frac{{\left( {1 + j} \right)\beta_{2} }}{{\beta_{1} }},1 + \frac{{\left( {1 + j} \right)\beta_{2} }}{{\beta_{1} }}, - \left( {\frac{1}{{\lambda_{1} }}} \right)^{{\beta_{1} }} } \right]}}{{\left( {1 + j} \right)\beta_{2} }}} \right] \\ & \quad + \left( {\begin{array}{*{20}c} { - \left( {\alpha_{2} + 1} \right)} \\ j \\ \end{array} } \right)\lambda_{2}^{{\beta_{2} }} \frac{{{\text{Hypergeometric}}2{\text{F}}1\left[ {\alpha_{1} ,\frac{{ - 1 + \alpha_{1} \beta_{1} + \left( {j + \alpha_{2} } \right)\beta_{2} }}{{\beta_{1} }},\frac{{ - 1 + \left( {1 + \alpha_{1} } \right)\beta_{1} + \left( {j + \alpha_{2} } \right)\beta_{2} }}{{\beta_{1} }}, - \left( {\frac{1}{{\lambda_{1} }}} \right)^{{ - \beta_{1} }} } \right]\left( {\left( {\frac{1}{{\lambda_{1} }}} \right)^{{ - \beta_{1} }} } \right)^{{\frac{{ - 1 + \alpha_{1} \beta_{1} + \left( {j + \alpha_{2} } \right)\beta_{2} }}{{\beta_{1} }}}} \left( {\left( {\frac{1}{{\lambda_{1} }}} \right)^{{\beta_{1} }} } \right)^{{\frac{{ - 1 + \left( {j + \alpha_{2} } \right)\beta_{2} }}{{\beta_{1} }}}} }}{{ - 1 + \alpha_{1} \beta_{1} + \left( {j + \alpha_{2} } \right)\beta_{2} }}. \\ \end{aligned}$$

#### *Proof*

$$\varvec{R} = P\left( {X < Y} \right) = \alpha_{1} \beta_{1} \lambda_{1}^{{\alpha_{1} }} \alpha_{2} \beta_{2} \lambda_{2}^{{\alpha_{2} }} \int\nolimits_{0}^{\infty } \int\nolimits_{0}^{y} x^{{\beta_{1} - 1}} \left( {\lambda_{1} + x^{{\beta_{1} }} } \right)^{{ - \alpha_{1} - 1}} y^{{\beta_{2} - 1}} \left( {\lambda_{2} + y^{{\beta_{2} }} } \right)^{{ - \alpha_{2} - 1}} dxdy$$.

 After some calculations

$$\varvec{R} = \alpha_{1} \beta_{1} \lambda_{1}^{{\alpha_{1} }} \left[ {1 - \lambda_{1}^{{\alpha_{1} }} \int \nolimits_{0}^{\infty } y^{{\beta_{2} - 1}} \left( {\lambda_{2} + y^{{\beta_{2} }} } \right)^{{ - \alpha_{2} - 1}} \left( {\lambda_{1} + y^{{\beta_{1} }} } \right)^{{ - \alpha_{1} }} dy} \right].$$

Using the expansion $$\left( {1 + x^{b} } \right)^{ - a} = \left\{ {\begin{array}{*{20}l} {\sum\nolimits_{j = 0}^{\infty } {\left( {\begin{array}{*{20}c} { - a} \\ j \\ \end{array} } \right)x^{{ - b\left( {j + a} \right)}} ;\quad \left| {x^{b} } \right| > 1} } \hfill \\ {\sum\nolimits_{j = 0}^{\infty } {\left( {\begin{array}{*{20}c} { - a} \\ j \\ \end{array} } \right)x^{bj} ;\quad \left| {x^{b} } \right| < 1} } \hfill \\ \end{array} } \right. ,$$ $$\left( {\begin{array}{*{20}c} { - a} \\ j \\ \end{array} } \right) = \left( { - 1} \right)^{j} \left( {\begin{array}{*{20}c} {a + j - 1} \\ j \\ \end{array} } \right).$$

 The following result has obtained

$$\varvec{R} = \alpha_{1} \beta_{1} \lambda_{1}^{{\alpha_{1}}} \left[{1 - \lambda_{2}^{{- \beta_{2} \left({\alpha_{2} + 1} \right)}}} \right]\lambda_{1}^{{\alpha_{1} \beta_{1}}} {\mathop \sum \limits_{j = 0}^{\infty} \left({\begin{array}{*{20}c} {- \left({\alpha_{2} + 1} \right)} \\ j \\ \end{array}} \right)} {\lambda_{2}^{{- \beta_{2} j}} \int \nolimits_{0}^{1} y^{{\beta_{2} \left({j + 1} \right) - 1}} \left({\left({1 + \frac{y}{{\lambda_{1}}}} \right)^{{\beta_{1}}}} \right)^{{- \alpha_{1}}}} {+ \left({\begin{array}{*{20}c} {- \left({\alpha_{2} + 1} \right)} \\ j \\ \end{array}} \right)\lambda_{2}^{{\beta_{2}}} \mathop \int\nolimits_{1}^{\infty} y^{{- \beta_{2} \left({j + \alpha_{2}} \right) - 1}}} \left(\left({1 + \frac{y}{{\lambda_{1}}}} \right)^{{\beta_{1}}^{{- \alpha_{1}}}} \right)dy$$

The integrals are then easy to determine and the proof is completed.

## Methods of estimation

In this section, we consider maximum likelihood estimation (MLE) to estimate the involved parameters and the method of moment estimates (MME). Moreover, the asymptotic distribution of $\hat{\Theta } = \left( {\hat{\alpha },\hat{\beta },\hat{\lambda }} \right)$ are obtained using the elements of the inverse Fisher information matrix.

### Maximum likelihood estimation

Let *x*_1_, *x*_2_, …, *x*_*n*_ be a random sample of size n from the POLO distribution with PDF given by Eq. (11)

The log-likelihood function (${\mathcal{L}}\left( {\alpha ,\beta ,\lambda } \right))$ of POLO distribution is given by

15 $$L\left( {\alpha ,\beta ,\lambda } \right) = n\left( {\ln \alpha + \ln \beta + \ln \lambda } \right) + \left( {\beta - 1} \right)\mathop \sum \limits_{i = 1}^{n} \ln x_{i} - \left( {\alpha + 1} \right)\mathop \sum \limits_{i = 1}^{n} \ln \left( {\lambda + x_{i}^{\beta } } \right)$$

It follows that the maximum likelihood estimators (MLEs), say $\hat{\alpha }$, $\hat{\beta }$ and $\hat{\lambda }$, are the simultaneous solutions of the equations

16 $$\frac{\partial }{\partial \alpha }L\left( {\alpha ,\beta ,\lambda } \right) = \frac{n}{\alpha } + n\ln \lambda - \mathop \sum \limits_{i = 1}^{n} \ln \left( {\lambda + x_{i}^{\beta } } \right)$$

17 $$\frac{\partial }{\partial \beta }L\left( {\alpha ,\beta ,\lambda } \right) = \frac{n}{\beta } + \mathop \sum \limits_{i = 1}^{n} \ln x_{i} - \left( {\alpha + 1} \right)\mathop \sum \limits_{i = 1}^{n} \frac{{x_{i}^{\beta } \ln x_{i} }}{{\left( {\lambda + x_{i}^{\beta } } \right)}}$$

18 $$\frac{\partial }{\partial \lambda }L\left( {\alpha ,\beta ,\lambda } \right) = \frac{n\alpha }{\lambda } - \left( {\alpha + 1} \right)\mathop \sum \limits_{i = 1}^{n} \frac{1}{{\left( {\lambda + x_{i}^{\beta } } \right)}}$$

### Method of moments

Let *x*_1_, *x*_2_, …, *x*_*n*_ be a random sample of size n from the POLO distribution with PDF given by Eq. (11), by equating the raw moments of POLO distribution with the sample moments, the MME equations are

$$\mu_{1}^{\prime} = \frac{{\alpha \lambda^{{\frac{1}{\beta }}} \varGamma \left[ {\alpha - \frac{1}{\beta }} \right]\varGamma \left[ {\frac{1}{\beta }} \right]}}{{\beta \varGamma \left[ {1 + \alpha } \right]}}.,\quad \mu_{2}^{\prime} = \frac{{\alpha \lambda^{2/\beta } \varGamma \left[ {\alpha - \frac{2}{\beta }} \right]\varGamma \left[ {\frac{2 + \beta }{\beta }} \right]}}{{\varGamma \left[ {1 + \alpha } \right]}}. ,\quad \mu_{3}^{\prime} = \frac{{\alpha \lambda^{3/\beta } \varGamma \left[ {\alpha - \frac{3}{\beta }} \right]\varGamma \left[ {\frac{3 + \beta }{\beta }} \right]}}{{\varGamma \left[ {1 + \alpha } \right]}}.$$

The method of moments estimators are the simultaneous solutions of these three equations.

### Fisher information matrix

For interval estimation of the parameter vector $\varTheta = \left( {\alpha ,\lambda ,\beta } \right)^{T}$ for POLO distribution; we can derive the expected Fisher information matrix ${\mathbf{I}} = \left[ {{\text{I}}_{\text{ij}} } \right]$, ${\text{i}},\,{\text{j}} = 1,2,3$ as follows:

$$\begin{aligned} I_{11}& = E\left[ {\frac{{ - \partial^{2} \text{lnf} \left( x \right)}}{{\partial \alpha^{2} }}} \right] = \frac{1}{{\alpha^{2} }}. \hfill \\ I_{22} &= E\left[ {\frac{{ - \partial^{2} \text{lnf} \left( x \right)}}{{\partial \lambda^{2} }}} \right] = \frac{\alpha \beta }{{\left( {2\beta + \alpha \beta } \right)\lambda^{2} }}. \hfill \\ I_{33} &= E\left[ {\frac{{ - \partial^{2} \text{lnf} \left( x \right)}}{{\partial \beta^{2} }}} \right] = \frac{1}{{\beta^{2} }} + \frac{{\lambda^{{ - \frac{2}{\beta }}} \left( {\lambda^{{\frac{1}{\beta }}} \varGamma \left[ {2 - \frac{1}{\beta }} \right]\varGamma \left[ {\alpha + \frac{1}{\beta }} \right] + \frac{{\alpha \left( { - 1 + \beta } \right)\beta \varGamma \left[ {2 - \frac{2}{\beta }} \right]\varGamma \left[ {\alpha + \frac{2}{\beta }} \right]}}{2 + \alpha }} \right)}}{\varGamma \left[ \alpha \right]}. \hfill \\ I_{12} &= E\left[ {\frac{{ - \partial^{2} \text{lnf} \left( x \right)}}{\partial \alpha \partial \lambda }} \right] = - \frac{\alpha \beta }{{\left( {\alpha \beta + \alpha^{2} \beta } \right)\lambda }}. \hfill \\ I_{23} &= E\left[ {\frac{{ - \partial^{2} \text{lnf} \left( x \right)}}{\partial \lambda \partial \beta }} \right] = - \frac{{\alpha \left( {1 + \alpha } \right)\beta \lambda^{{\alpha - \frac{1 + \beta + \alpha \beta }{\beta }}} \varGamma \left[ {2 - \frac{1}{\beta }} \right]\varGamma \left[ {1 + \alpha + \frac{1}{\beta }} \right]}}{{\varGamma \left[ {3 + \alpha } \right]}}. \hfill \\ I_{13} &= E\left[ {\frac{{ - \partial^{2} \text{lnf} \left( x \right)}}{\partial \alpha \partial \beta }} \right] = \frac{{\alpha \beta \lambda^{{\alpha - \frac{1 + \alpha \beta }{\beta }}} \varGamma \left[ {2 - \frac{1}{\beta }} \right]\varGamma \left[ {\alpha + \frac{1}{\beta }} \right]}}{{\varGamma \left[ {2 + \alpha } \right]}}. \hfill \\ \end{aligned}$$

Under regularity conditions, Bahadur (1964), showed that as $n \to \infty ,\sqrt n \left( {\hat{\varTheta } - \varTheta } \right)$ is asymptotically normal 3-variate with (vector) mean zero and covariance matrix ${\mathbf{I}}^{ - 1}$. Asymptotic variances and covariance of the elements of $\hat{\varTheta }$ are obtained by:

$$\text{var} \left( {\hat{\alpha }} \right) = \frac{{I_{22} I_{33} - I_{23}^{2} }}{n\Delta },\quad \text{var} \left( {\hat{\lambda }} \right) = \frac{{I_{11} I_{33} - I_{13}^{2} }}{n\Delta },\quad \text{var} \left( {\hat{\beta }} \right) = \frac{{I_{11} I_{22} - I_{12}^{2} }}{n\Delta }$$

$$\text{cov} \left( {\hat{\alpha },\hat{\lambda }} \right) = \frac{{I_{13} I_{23} - I_{12} I_{33} }}{n\Delta },\;\text{cov} \left( {\hat{\alpha },\hat{\beta }} \right) = \frac{{I_{12} I_{23} - I_{13} I_{22} }}{n\Delta },\;\text{cov} \left( {\hat{\lambda },\hat{\beta }} \right) = \frac{{I_{13} I_{12} - I_{11} I_{23} }}{n\Delta }$$

where $\Delta = { \det }\left( {\mathbf{I}} \right)$. The corresponding asymptotic $100\left( {1 - \alpha } \right)\%$ confidence intervals are $\hat{\Theta } \pm {\text{c}} {{\mathbf{I}}^{- 1/2}}$; where c is the appropriate z critical value.

## Simulation studies

The Equation $F\left( x \right) - u = 0$, where u is an observation from the uniform distribution on (0,1) and *F*(*x*) is cumulative distribution function of distribution is used to carry out the simulation study to generate data from distribution. The simulation experiment was repeated *N* = 1000 times each with sample sizes; *n* = 30, 50, 70, 90 *and* (*α*, *β*, *λ*) = (0.5, 10, 0.5), (0.5,5,1). The following measures are computed.

Average bias and the mean square error (MSE) of $\hat{\gamma }$ of the parameter *α*, *β*, *λ*

$$\frac{1}{N}\mathop \sum \limits_{i = 1}^{N} (\hat{\gamma } - \gamma ) \quad \frac{1}{N}\mathop \sum \limits_{i = 1}^{N} \left( {\hat{\gamma } - \gamma } \right)^{2}$$

Table 2 presents the average bias and the MSE of the estimates. The values of the bias and the MSEs decreases while the sample size increases.

**Table 2:** Bias and MSE for the POLO parameters

*α*	*β*	*λ*	*n*	Bias (*α*)	MSE (*α*)	Bias (*β*)	MSE (*β*)	Bias (*λ*)	MSE (*λ)*
0.5	10	0.5	30	0.1508	0.03394	2.7765	19.9278	0.7227	11.9722
			50	0.1394	0.0265	2.4762	12.6226	0.1764	4.6215
			70	0.1337	0.0228	2.3086	10.4639	0.0368	1.9079
			90	0.1319	0.0209	2.0271	7.2067	0.0872	1.4503
0.5	5	1	30	0.1358	0.0205	1.1525	2.1615	0.2566	0.8045
			50	0.1279	0.0175	1.0211	1.4909	0.2959	0.3731
			70	0.1247	0.0163	0.9626	1.2279	0.3059	0.1603
			90	0.1246	0.0162	0.8856	1.0043	0.3333	0.1527

## Application

Consider a dataset corresponding to remission times (in months) of a random sample of 128 bladder cancer patients given in Lee and Wang (2003). The data are given as follows: 0.08, 2.09, 3.48, 4.87, 6.94, 8.66, 13.11, 23.63, 0.20, 2.23, 3.52, 4.98, 6.97, 9.02, 13.29, 0.40, 2.26, 3.57, 5.06, 7.09, 9.22, 13.80, 25.74, 0.50, 2.46, 3.64, 5.09, 7.26, 9.47, 14.24, 25.82, 0.51, 2.54, 3.70, 5.17, 7.28, 9.74, 14.76, 26.31, 0.81, 2.62, 3.82, 5.32, 7.32, 10.06, 14.77, 32.15, 2.64, 3.88, 5.32, 7.39, 10.34, 14.83, 34.26, 0.90, 2.69, 4.18, 5.34, 7.59, 10.66, 15.96, 36.66, 1.05, 2.69, 4.23, 5.41, 7.62, 10.75, 16.62, 43.01, 1.19, 2.75, 4.26, 5.41, 7.63, 17.12, 46.12, 1.26, 2.83, 4.33, 5.49, 7.66, 11.25, 17.14, 79.05, 1.35, 2.87, 5.62, 7.87, 11.64, 17.36, 1.40, 3.02, 4.34, 5.71, 7.93, 11.79, 18.10, 1.46, 4.40, 5.85, 8.26, 11.98, 19.13, 1.76, 3.25, 4.50, 6.25, 8.37, 12.02, 2.02, 3.31, 4.51, 6.54, 8.53, 12.03, 20.28, 2.02, 3.36, 6.76, 12.07, 21.73, 2.07, 3.36, 6.93, 8.65, 12.63, 22.69.

We have fitted the Power Lomax distribution to the dataset using MLE, and compared the proposed Power Lomax distribution with Lomax, MCLomax, BLomax, KW Lomax, exponential Lomax, G-lomax, transmuted exponentiated Lomax, WLomax, extended Poisson Lomax and ELomax. The model selection is carried out using the AIC (Akaike information criterion), the BIC (Bayesian information criterion), the CAIC (consistent Akaike information criteria) and the HQIC (Hannan Quinn information criterion).

19 $$\begin{aligned} AIC &= - 2L\left( {\hat{\theta }} \right) + 2q, \hfill \\ BIC &= - 2L\left( {\hat{\theta }} \right) + q\log \left( n \right), \hfill \\ HQIC &= - 2L\left( {\hat{\theta }} \right) + 2q\log (\log \left( n \right)) , \hfill \\ CAIC &= - 2L\left( {\hat{\theta }} \right) + \frac{2qn}{{\left( {n - q - 1} \right)}} \hfill \\ \end{aligned}$$

where $L\left( {\hat{\theta }} \right)$ denotes the log-likelihood function evaluated at the maximum likelihood estimates, q is the number of parameters, and *n* is the sample size. Here we let *θ* denotes the parameters, i.e., $\theta = \left( {\alpha ,\beta ,\lambda } \right).$ An iterative procedure is applied to solve Eqs. (16), (17) and (18) and consequently obtain $\hat{\theta } = \left( {\hat{\alpha } = 2.07012 ,\;\hat{\beta } = 1.4276,\;\hat{\lambda } = 34.8626} \right)$. At these values we calculate the log-likelihood function given by (15) and apply relation (19). The model with minimum AIC (or BIC, CAIC and HQIC) value is chosen as the best model to fit the data. From Table 3, we conclude that the Power Lomax distribution is best comparable to the Lomax, MCLomax, BLomax, KW Lomax, exponential Lomax (Exp.Lomax), G-lomax, transmuted exponentiated Lomax (TE-Lomax), WLomax, extended Poisson Lomax (Ext.PLD) and ELomax distributions.

**Table 3:** MLEs and the measures AIC, BIC, HQIC and CAIC

Distribution	Estimates	−Log L	AIC	BIC	HQIC	CAIC
Lomax	$\hat{\alpha } = 13.9384$ $\hat{\lambda } = 121.023$	−413.835	831.67	837.37	833.98	831.80
MCLomax	$\hat{\alpha } = 0.8085$ $\hat{\beta } = 11.2929$ $\hat{a} = 1.5060$ $\hat{\eta } = 4.1886$ $\hat{c} = 2.1046$	−409.91	829.82	844.09	835.62	830.14
BLomax	$\hat{\alpha } = 3.9191$ $\hat{\beta } = 23.9281$ $\hat{a} = 1.5853$ $\hat{\eta } = 0.1572$	−411.743	831.486	842.89	836.12	831.74
KW Lomax	$\hat{\alpha } = 0.3911$ $\hat{\beta } = 12.2973$ $\hat{a} = 1.5162$ $\hat{\eta } = 11.0323$	−409.94	827.88	839.29	832.52	828.14
Exp Lomax	$\hat{\alpha } = 1.0644$ $\hat{\beta } = 0.08$ $\hat{\lambda } = 0.006$	−414.978	835.956	844.512	839.432	836.15
G-lomax	$\hat{\alpha } = 4.754$ $\hat{\beta } = 20.581$ $\hat{a} = 1.5858$	−410.081	826.162	834.718	829.638	826.36
TE-Lomax	$\hat{\alpha } = 1.71418$ $\hat{\gamma } = 0.05456$ $\hat{\lambda } = 0.24401$ $\hat{\theta } = 3.33911$	−410.434	828.868	840.276	833.505	829.13
WLomax	$\hat{\alpha } = 0.25661$ $\hat{\beta } = 1.57945$ $\hat{a} = 2.42151$ $\hat{b} = 1.86389$	−410.811	829.622	841.03	834.257	829.88
Ext.PLD	$\hat{\alpha } = 0.2387$ $\hat{\beta } = 8.04 \times 10^{ - 3}$ $\hat{\lambda } = 59.8378$	−413.835	833.67	842.22	837.14	833.86
ELomax	$\hat{\alpha } = 4.5857$ $\hat{\beta } = 24.7414$ $\hat{a} = 1.5862$	−410.07	826.14	834.70	829.62	826.33
Power Lomax	$\hat{\alpha } = 2.07012$ $\hat{\beta } = 1.4276$ $\hat{\lambda } = 34.8626$	−409.74	825.48	834.036	828.956	825.67

For an ordered random sample, *X*_1_, *X*_2_, …, *X*_*n*_,  from Power Lomax distribution (*α*, *β*, *λ*), where the parameters *α*, *β* and *λ* are unknown, the Kolmogorov–Smirnov *D*_*n*_, Cramér-von Mises *W*_*n*_^2^, Anderson and Darling *A*_*n*_^2^, Watson *U*_*n*_^2^ and Liao-Shimokawa *L*_*n*_^2^ tests statistics are given as follows (For details see e.g. Al-Zahrani 2012)

$$D_{n} = \mathop {\hbox{max} }\limits_{i} \left( {\frac{i}{n} - F\left( {x_{i} ,\hat{\alpha },\hat{\beta },\hat{\lambda }} \right),F\left( {x_{i} ,\hat{\alpha },\hat{\beta },\hat{\lambda }} \right) - \frac{i - 1}{n}} \right)$$

$$W_{n}^{2} = \frac{1}{12n} + \mathop \sum \limits_{i = 1}^{n} \left( {F\left( {x_{i} ,\hat{\alpha },\hat{\beta },\hat{\lambda }} \right) - \frac{2i - 1}{n}} \right)^{2}$$

$$A_{n}^{2} = - n - \mathop \sum \limits_{i = 1}^{n} \frac{2i - 1}{n}\left[ {\ln \left( {F\left( {x_{i} ,\hat{\alpha },\hat{\beta },\hat{\lambda }} \right)} \right) + \ln \left( {1 - F\left( {x_{i} ,\hat{\alpha },\hat{\beta },\hat{\lambda }} \right)} \right)} \right]$$

$$U_{n}^{2} = W_{n}^{2} + \mathop \sum \limits_{i = 1}^{n} \left( {\frac{{F\left( {x_{i} ,\hat{\alpha },\hat{\beta },\hat{\lambda }} \right)}}{n} - \frac{1}{2}} \right)^{2}$$

$$L_{n} = \frac{1}{\sqrt n }\mathop \sum \limits_{i = 1}^{n} \frac{{\mathop {\hbox{max} }\limits_{i} \left[ {\frac{i}{n} - F\left( {x_{i} ,\hat{\alpha },\hat{\beta },\hat{\lambda }} \right),F\left( {x_{i} ,\hat{\alpha },\hat{\beta },\hat{\lambda }} \right) - \frac{i - 1}{n}} \right]}}{{\sqrt {F\left( {x_{i} ,\hat{\alpha },\hat{\beta },\hat{\lambda }} \right)\left[ {1 - F\left( {x_{i} ,\hat{\alpha },\hat{\beta },\hat{\lambda }} \right)} \right]} }}$$

Table 4 indicates that the test statistics *D*_*n*_, $W_{n}^{2}$, $A_{n}^{2}$, $U_{n}^{2}$ and L_*n*_ have the smallest values for the data set under Power Lomax distribution model with regard to the other models. The proposed model offers a smart alternative to the above distributions. The Power Lomax distribution approximately provides an adequate fit for the data.

**Table 4:** Goodness-of-fit tests

Distribution	*D*_*n*_	$\varvec{W}_{\varvec{n}}^{2}$	$\varvec{A}_{\varvec{n}}^{2}$	$\varvec{U}_{\varvec{n}}^{2}$	*L*_*n*_
Lomax	0.096669	0.2125894	1.374568	31.70173	1.059358
MCLomax	0.039118	0.0226833	0.157007	31.52438	0.447921
BLomax	0.040501	0.0258228	0.178724	31.52722	0.475089
KW Lomax	0.038908	0.0229529	0.159531	31.52472	0.451879
Exp.Lomax	0.076702	0.1796769	1.0908	31.69346	1.084015
G-lomax	0.040639	0.0261905	0.18089	31.52988	0.477688
TE-Lomax	0.03991	0.0314384	0.227535	31.53149	0.534136
WLomax	0.041403	0.0382951	0.262735	31.54341	0.577467
Ext.PLD	0.098877	0.2267612	1.451105	31.7139	1.073414
ELomax	0.039863	0.02682087	0.18341	31.52886	0.483202
Power Lomax	0.035055	0.01754725	0.120466	31.51976	0.404336

**Fig. 5:**
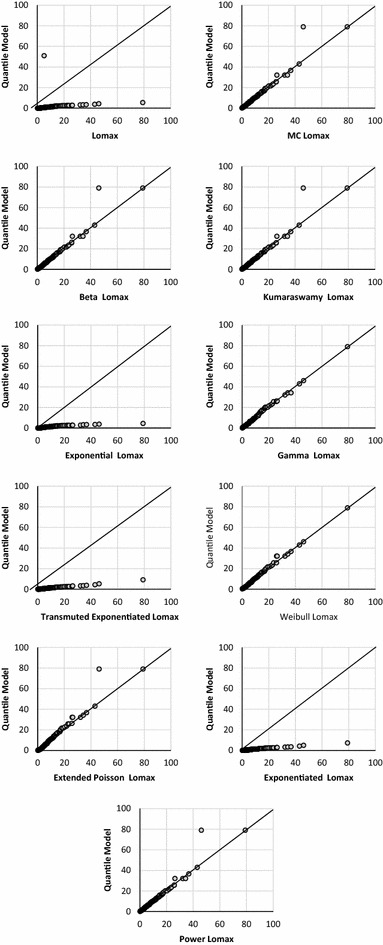
The Q–Q plot for bladder cancer data

The quantile–quantile or Q–Q plot is used to check the validity of the distributional assumption for the data. Figure 5 shows that the data seems to follow a Power Lomax distribution reasonably well, except some points on extreme.

## Conclusion

In this paper we introduced a three parameters power Lomax Distribution (POLO). The new distribution can exhibit a much more flexible model for life time data especially bladder cancer data than its predecessor Lomax distributions, presenting decreasing, inverted bath tub hazard rate function. Most statistical and reliability properties are derived and studied. Simulation schemes are formulated and provides less bias and mean square error as sample size increases for MLEs of POLO parameters. Point Estimation via MME and MLE methods are done moreover, the Fisher information matrix for interval estimation is studied for POLO. A real data on bladder cancer is used to illustrate and compare the potential of POLO distribution with other competing distributions showed that it could offer a better fit than a set of extensions of Lomax distribution.
